# Robotic Pelvic Exenteration for Gynecologic Malignancies, Anatomic Landmarks, and Surgical Steps: A Systematic Review

**DOI:** 10.3389/fsurg.2021.790152

**Published:** 2021-11-30

**Authors:** Stefano Cianci, Martina Arcieri, Giuseppe Vizzielli, Canio Martinelli, Roberta Granese, Marco La Verde, Anna Fagotti, Francesco Fanfani, Giovanni Scambia, Alfredo Ercoli

**Affiliations:** ^1^Department of Human Pathology of Adult and Childhood "G. Barresi", Unit of Gynecology and Obstetrics, University of Messina, Messina, Italy; ^2^Department of Biomedical, Dental, Morphological, and Functional Imaging Sciences, University of Messina, Messina, Italy; ^3^Department of Obstetrics Gynecology and Pediatrics, University of Udine, Udine, Italy; ^4^Department of Woman, Child and General and Specialized Surgery, University of Campania "Luigi Vanvitelli", Caserta, Italy; ^5^Division of Gynecologic Oncology, Fondazione Policlinico Universitario A. Gemelli IRCCS, Rome, Italy; ^6^Institute of Obstetrics and Gynecology, Universita' Cattolica del Sacro Cuore, Rome, Italy

**Keywords:** anatomy, pelvic exenteration, gynecological cancer, robotic surgery, minimally invasive surgery

## Abstract

Pelvic exenteration represents the last resort procedure for patients with advanced primary or recurrent gynecological malignancy. Pelvic exenteration can be divided into different subgroup based on anatomical extension of the procedures. The growing application of the minimally invasive surgical approach unlocked new perspectives for gynecologic oncology surgery. Minimally invasive surgery may offer significant advantages in terms of perioperative outcomes. Since 2009, several Robotic Assisted Laparoscopic Pelvic Exenteration experiences have been described in literature. The advent of robotic surgery resulted in a new spur to the worldwide spread of minimally invasive pelvic exenteration. We present a review of the literature on robotic-assisted pelvic exenteration. The search was conducted using electronic databases from inception of each database through June 2021. 13 articles including 53 patients were included in this review. Anterior exenteration was pursued in 42 patients (79.2%), 2 patients underwent posterior exenteration (3.8%), while 9 patients (17%) were subjected to total exenteration. The most common urinary reconstruction was non-continent urinary diversion (90.2%). Among the 11 women who underwent to total or posterior exenteration, 8 (72.7%) received a terminal colostomy. Conversion to laparotomy was required in two cases due to intraoperative vascular injury. Complications' report was available for 51 patients. Fifteen Dindo Grade 2 complications occurred in 11 patients (21.6%), and 14 grade 3 complications were registered in 13 patients (25.5%). Only grade 4 complications were reported (2%). In 88% of women, the resection margins were negative. Pelvic exenteration represents a salvage procedure in patients with recurrent or persistent gynecological cancers often after radiotherapy. A careful patient selection remains the milestone of such a mutilating surgery. The introduction of the minimally invasive approach has led to advantages in terms of perioperative outcomes compared to classic open surgery. This review shows the feasibility of robotic pelvic exenteration. An important step forward should be to investigate the potential equivalence between robotic approaches and the laparotomic one, in terms of long-term oncological outcomes.

## Introduction

Pelvic exenteration represents the last resort procedure for patients with advanced primary or recurrent gynecological malignancy, particularly in those with irradiated pelvis and thus unsuitable for further radiotherapy (1). The first pelvic exenteration in the literature dates back to 1948 (2). Pelvic exenteration consists in the partial or total removal of the pelvic structures.

Anterior pelvic exenteration includes partial or total excision of the vagina, removal of the genital organs and bladder and eventually partial or total excision of the urethra and is performed in patients with malignancies secondarily involving the bladder. Posterior pelvic exenteration includes partial or total excision of the vagina, removal of the genital organs and sigma rectum and is performed in patients with malignancies involving the rectum. Total pelvic exenteration comprises partial or total excision of the vagina, removal of the rectum, genital organs and bladder and eventually partial or total excision of the urethra. Therefore, three radical monovisceral surgical operations (resection of the rectum, hysterocolpectomy, and cystectomy) are combined in pelvic exenteration. Often, the recurrent disease infiltrates beyond the adjacent pelvic viscera involving different anatomical structures out of the “pelvic box”; such as bones, muscles and neurovascular components of pelvic lateral wall. Therefore, pelvic exenterative surgery must be tailored on tumor tridimensional topography. In view of this, a correct classification should consider not only anterior, posterior, or total extension but also resection of lateral pelvic side wall and levator ani muscle (3–6). According to the extent of the surgery, pelvic exenteration is classified as type I (supralevator, preserving endopelvic fascia and pelvic diaphragm), type II (infralevator, including resection of levator ani muscle), or type III (infralevator with vulvectomy) (7). In type IIA PE, the urogenital diaphragm and distal vagina are preserved while type IIB PE includes total vaginectomy and resection of both pelvic and urogenital diaphragms. Type I and IIA posterior exenteration usually could allow to make a colorectal anastomosis (8). The extent of the vulva excision depends on the tumor location and ranges from no removal to extensive excision of skin and soft tissue from the perineal and perianal area. When the tumors are fixed to the lower pelvic side wall, laterally extended endopelvic resection is necessary to achieve tumor-free resection margins (9). This procedure is characterized by the removal of the complete pelvic visceral compartments en bloc with one or more of these endopelvic parietal structures: paravisceral fat pad, internal iliac vessels, obturator internus muscle, and pubococcygeus, iliococcygeus, and coccygeus muscles (10). Therefore, the aim of pelvic exenteration is to achieve tumor-free resection margins, increasing survival, with lower surgical-associated morbidity. Thanks to the improvements in surgical technique, technology, and perioperative care, it has been observed a progressive shift in the purpose of this aggressive surgery from exclusively palliative to a curative one. As a result, 5-years overall survival after PE is increased reaching 40% (11).

The growing application of the minimally invasive surgical approach, particularly in urologic and oncological colorectal surgery, unlocked new perspectives for gynecologic oncology surgery. Subsequently, the advent of robotic surgery resulted in a new spur to the worldwide spread of minimally invasive pelvic exenteration. The first case of robot-assisted laparoscopic pelvic exenteration was reported in 2009, when Lim (12) performed a total evisceration with an ileal loop urinary diversion, reporting no postoperative complications. Since then, several Robotic Assisted Laparoscopic Pelvic Exenteration experiences have been described in literature. The magnified 3-dimensional vision and the articulated movement of robotic instruments allow to perform complex surgical procedures even in the narrow spaces of the pelvis, and in challenging retroperitoneal condition such as in obese or radio-treated patients (13, 14). To deepen this topic, in this article, we present a review of the literature on robotic-assisted pelvic exenteration.

## Materials and Methods

**Figure 1:**
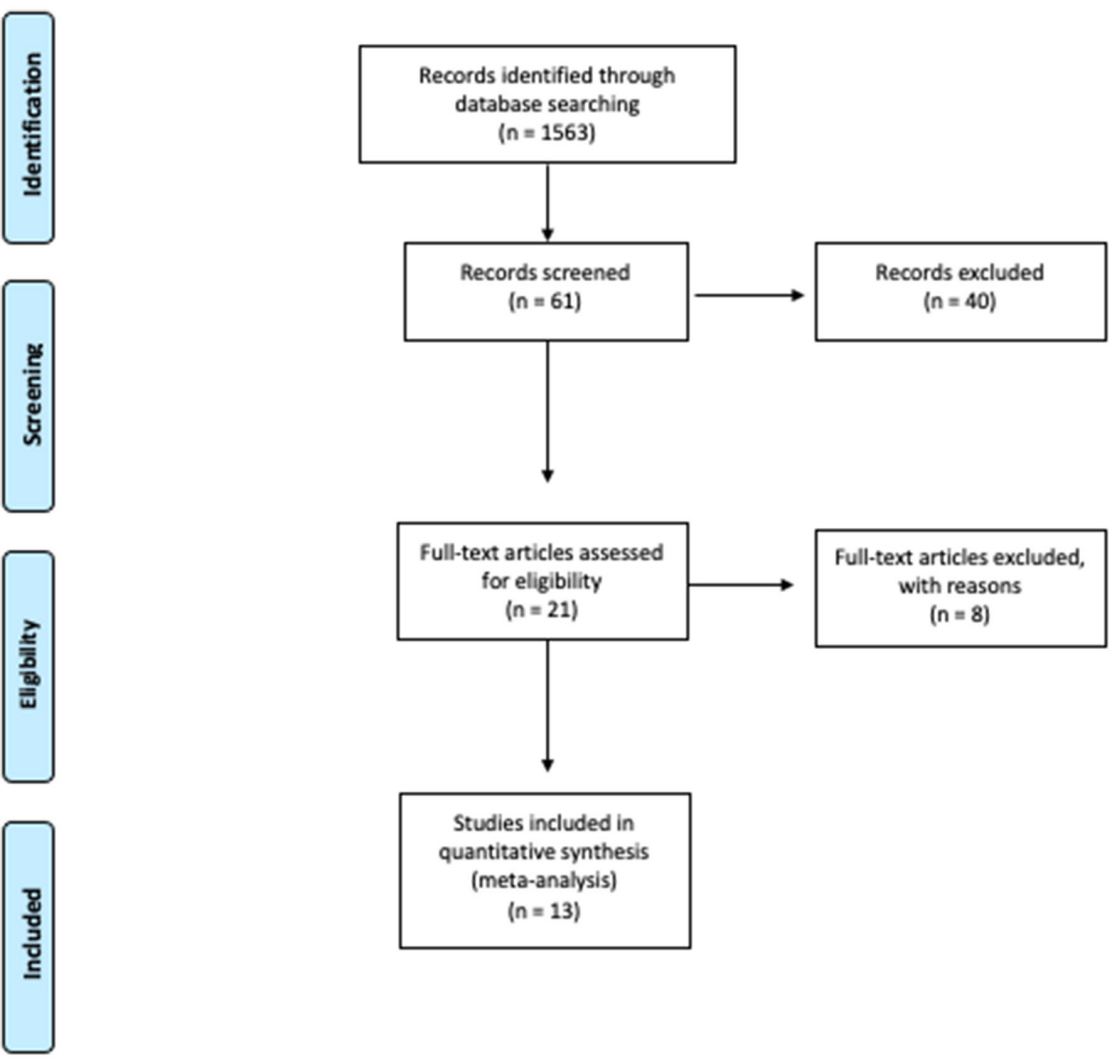
Study flow chart (15). For more informations, available online at: www.prisma-statement.org.

Preliminary research was conducted using the PubMed and SCOPUS databases. The PRISMA based flow-diagram in Figure 1 depicts our search strategy.

The search strategy applied in both databases included combinations of the keywords (“robot” OR “robotic”) AND “pelvic exenteration” AND “cervical cancer” AND “endometrial cancer” AND “ovarian cancer” AND “vulvar cancer.” The references for the relevant article were also hand-searched. Exclusion criteria included duplicate articles, papers non-pertinent with the current topic, particularly those regarding robotic pelvic exenteration for non-gynecological malignancies, commentary, letters to the editor. Two authors (MA and RG) screened each study independently and matched by title and abstract. Full texts of eligible studies were reviewed by two authors for data extraction. A third independent author (SC) supervised the research.

## Results

### Study Selection

After the first search, a total of 1,563 titles were extracted from the Pubmed and Scopus database, using the above mentioned keywords. After the first revision, 61 studies were extracted.

After matched revision, 21 articles were selected, of which 13 studies were ultimately eligible for the present review after full text evaluation. The selection diagram is reported in Figure 1.

### Synthesis of Results

At the time of the analysis (June 2021), 13 articles have been published on robotic pelvic exenterations, reporting a total of 53 patients.

The main findings of these studies are summarized in Table 1. The 13 articles include one published in French and 12 published in English.

**Table 1:** General characteristics of the included studies.

**N**	**References**	**N. cases**	**Primary Tumor Site**	**Indication**	**PE classification**	**Type of urinary reconstruction**	**Type of bowel recons truction**	**Intraoperative complications**	**Conversion**	**Median op time**	**Median EBL**	**Hospital stay**	**Postop complications (16)**	**Postop mortality**	**Margin of resection**	**Survival**
1	Lim (12)	1	Cervix	Recurrence after RTCT	Total	Ileal conduit	End colostomy	0	0	375	375	10	0	0	NR	NR
2	Lambaudie et al. (17)	3	Cervix	2 Recurence after surgery + RT, 1 Rec after RT	Anterior	Miami Pouch	NA	0	0	480	400	30	pt.1: urocutaneous fistula (G3), pt.2: ureteral stenosis (G3)	0	3 R0	NR
3	Davis et al. (18)	2	Cervix	1 Recurrence after CTRT, 1 Rec after RT	Anterior	Ileal conduit	NA	0	0	540	550	8	NR	NR	2 R0	NR
4	Vasilescu et al. (19)	1	Endometrium	Recurrence after surgery + RT	Total	Ureterostomy	End colostomy	0	0	250	365	11	0	0	NR	NR
5	Jauffret et al. (20)	2	Cervix	Recurrence	Anterior	Miami Pouch	NA	0	0	480	300	NR	pt.1: wound dehiscence (G2), urocutaneous fistula (G3), pt.2: CVC sepsis (G2), urosespis (G2), 1 prerenal failure (G2), obstructive renal failure (G3)	0	1 R0 1 R1	1. PFS 8 mo 23 mo OS, 2. 5 PFS, 22 OS
6	Lawande et al. (21)	1	Cervix	Primary	Total	Wet colostomy	End Colostomy	0	0	250	365	11	0	0	NR	NR
7	Puntambekar et al. (22)	10	Cervix	6 Primary, 2 Recurrence, 2 Vescico-vaginal fistula	Anterior	Ileal conduit	NA	0	0	180	110	5	0	0	10 R0	11 mo median FUP: 1 died 7 mo later for hepatic recurrence, 1 paraortic and hepatic recurrence after 6 mo, 8 NED
8	Kostantinidis et al. (23)	1	Cervix	Recurrence after RTCT	Total	Ileal conduit	End colostomy	0	0	641	400	NR	NR	NR	1 R0	NR
9	Kim et al. (24)	1	Melanoma	Recurrence after surgery + CT	Anterior	Ileal conduit	NA	0	0	270	NR	NR	0	0	1 R0	NED at 9 mo
10	Nguyen Xuan et al. (25)	5	Cervix	1 Recurrence after RTCT, 4 Recurrence after surgery + RTCT	2 Anterior, 1 Total, 2 Posterior	3 Bricker	2 colorectal anastomoses 1 end colostomy	0	0	402	NR	11.5	pt.1: UTI (G2), wound dehiscence (G2), pt.2: sepsis (G2), anastomosis stenosis (G3), pt.3 UTI (G2), acute obstructive renal failure (G3), pt.4: TPE (G2), pt.5: UTI (G2)	0	4 R0 1 R1	3 recurrences at 6, 7 and 7 mo
11	Yang et al. (26)	1	Cervix	Recurrence after surgery + RTCT	Total	Bricker's ileal conduit	Colorectal anastomosis	0	0	700	300	37	Rectal anastomosi leak (G3)	0	1 R0	NED at 17 mo
12	Bizzarri et al. (27)	11	2 endometrium, 8 cervix, 1 urothelial	8 Recurrence 2 Persistence 1 Primary	8 Anterior 3 Total	Ileal conduit	End colostomy	2 vascular lesion	2	500	235	9	3 late G3 complications (NR complications < G3)	0	7 R0 4 R1	Not available for robotic surgery only
13	Jain et al. (28)	14	Cervix	10 Recurrence, 4 Persistence	Anterior	Ileal conduit	NA		0	305	135	6.5	pt.1: prolapsed stoma (G1), pt.2: urosepsis (G2), UTI (G2), pt.3: large bowel obstruction (G3); pt.4: subacute intestinal obstruction (G2); pt.5: sigma perforation and peritonitis (G4); pt.6: recto-vaginal fistula (G3); pt.7: uretero-ileal anastamotic leak (G3); pt.8: paralytic ileus (G2); pt.9: bleeding from ileal conduit (G2); pt.10: ureteral stenosis bilateral (G3)	1	14 R0	5 pts died (4 for recurrence, 1 for postop complications). 7 NED. Median time to death: 12 months. 12-month DFS: 68.2% 12-month OS: 77.1%.

*PE, pelvic exenteraion; EBL, estimated blood loss; Postop, postoperative; Pt, patient; RT, radiotherapy; CT, chemotherapy; RTCT, concurrent radiotherapy and chemotherapy; UTI, urinary tract infection; NR, not reported; NED, non-evidence disease*.

Anterior exenteration was pursued in 42 patients (79.2%), 2 patients underwent posterior exenteration (3.8%), while 9 patients (17%) were subjected to total exenteration.

Urinary reconstruction was performed with a continent urinary diversion in 5 patients (9.8%) and with a non-continent urinary diversion in 46 patients (90.2%), including 44 ileal conduit urinary diversions, 1 wet colostomy, and 1 ureterostomy.

Among the 11 women who underwent to total or posterior exenteration, 3 (27.3%) were primary re-anastomized, and 8 (72.7%) received a terminal colostomy.

Intraoperative complications occurred in 2 women (5%) due to vascular injury. Both cases required a conversion to laparotomy.

In two articles postoperative complications were not reported, so complications report was available for 51 patients.

Fifteen Dindo et al. (16) Grade 2 complications occurred in 11 patients (21.6%), and 14 grade 3 complications were registered in 13 patients (25.5%). Only grade 4 complications were reported (2%).

A complete surgical cytoreduction with negative resection margins was achieved in 44 patients (88%) while a suboptimal cytoreduction was reached in 6 patients (12%). For three patients 5.7%), there was no pathologic report.

Data regarding follow up were available for 31 patients (from 6 out of 13 studies included).

As reported in these studies, 19 (61.3%) patients were disease free (NED), 8 (25.8%) were dead (7 death of disease, 1 death of postoperative complication), and 4 (12.9%) were alive with disease (AWD) at 12 months of median follow up.

## Discussion

### Indication for Pelvic Exenteration

The indication for pelvic evisceration is one of the most hotly debated topics and it must flow from the assessment of multiple factors to avoid inappropriate treatment.

In particular, previous treatments, presence of metastatic disease, infiltration of unresectable structures, general health conditions, nutritional status, and socioeconomic bedrock must be considered in order to address each patients toward the best therapeutic option (29–31).

Peritoneal carcinosis, distant metastases and invasion in the sciatic nerve and sacral plexus are considered as contraindications for pelvic exenteration (32).

The presence of lymph node involvement is still a controversial issue. Although some studies identify it as an adverse prognostic factor (33, 34), others do not consider the presence of pelvic or aortic lymph node metastases as a contraindication to pelvic exenteration, especially in the setting of palliative surgery (35–38).

The first pelvic eviscerations were performed on recurrences of cervical carcinoma; then, the indication was extended to all gynecological neoplasms (endometrial, vulvar, ovarian carcinoma). However, as reported in the published literature (10) and in this review, the most common indication remains recurrent or persistent cervical carcinoma. The 5-year survival reported was about 40–50% for patients with recurrent or persistent tumor that does not exceed 5 cm and with free-tumor margins (10).

As reported in this review, robotic pelvic exenteration was performed in most cases in patients who had undergone previous treatments (radiotherapy, surgery, chemotherapy, or combined treatments), only eight (15.1%) were naïve patients.

### Surgical Techniques

To achieve free surgical margins PE surgical steps must be tailored to the specific case, evaluating tumor localization, the involvement of pelvic organs and structures and previous treatment, including pelvic radiation. The surgeon adjusts the surgical procedures according to intraoperative findings but there are some essentials surgical steps. PE can be divided into three surgical phases: explorative; ablative; and reconstructive.

The first step is the exploration of the abdominal and pelvic cavity to exclude the presence of metastatic peritoneal disease or liver metastasis. Lateral resectability is assessed during this phase, and in case of massive pelvic wall involvement, the surgery could be aborted.

The goals of the demolition phase are to obtain free margins and to decrease the surgery-associated morbidity. This phase starts with paravescial and Latzko and Okabayashi pararectal spaces development to identify and to dissect the pelvic neurovascular structure and the ureter. Umbilical and uterine artery are dissected and closed at their origin. The dissection is continued caudally in the paravesical space to reach the levator ani muscle. During total or anterior PE, Retzius space is opened ventrally, and the bladder was dissected off the anterior abdominal wall. In case of posterior PE the bladder is dissected completely from the cervix and from the proximal part of the vagina. After colon mobilization, the presacral space is opened (posterior and total PE) while during anterior PE, the rectovaginal space is developed, and the rectum is dissected from the vagina. Subsequently, the surgeon performs the parametrial resection. After section of the umbilical and uterine artery and isolation and section of the deep uterine vein, lateral parametria are resected at the lateral pelvic attachments. In a total or anterior PE, the ureters are resected above their involvement in the tumor, often with an intraoperative assessment of margins. Ventral parametria are transected at the posterior bladder wall (posterior PE) or at the attachment to the inferior portion of the pubic bones (total or anterior PE). The surgeon resects the dorsal parametria at the level of the rectum (anterior PE) or on the presacral space at the level of sacral bone (total or posterior PE). After the resection of the parametria, the exenteration specimen is mobilized centrally in the pelvis. The sigmoid is then resect caudally (posterior or total PE). At this point, steps diverge depending on whether supralevator or infralevator exenteration is planned. Supralevator exenteration finishes with removal of the specimen above the levator muscles. The rectum is resected caudally at the level of the pelvic floor (total and posterior PE); urethra is transected (total and anterior PE) and the vagina are resected below the level of the tumor with adequate margins. These completes detachment of the specimen, which includes bladder, uterus, rectum, and surrounding tissue.

In case of infralevator PE (types II and III), the pelvic floor is resected. A total vaginectomy may be part of infralevator PE. A total vaginectomy and urethrectomy (total or anterior PE) is obtained through a circumferential incision inside the vulva; if necessary, resection of the anus is also made. The vagina is dissected off the elevator muscles unless they have tumor involvement. In this case, the muscle is excised to obtain free margin. In case of total or posterior PE, the rectum is resected caudally at the level of the middle vagina (if a complete perineal resection is not necessary), and the specimen is removed en-block.

The preservation of a distal part of the vagina and the urogenital diaphragm improves the quality of sexual life and decreases the risk of empty pelvis syndrome. In case of vulva or distal part of the vagina or rectovaginal septum involvement, distal colpectomy or vulvectomy, or both are performed from a perineal approach. During type I PE, the specimen is removed abdominally while after infralevator exenteration it can be removed either vaginally or abdominally (8).

The last phase is the reconstructive one. If the anal sphincter cannot be spared in cases of type II-III PE, the feces are diverted trough permanent end colostomy. During type I PE with anal sphincter sparing, bowel continuity could be restored by a deep colorectal or coloanal anastomosis, if technically feasible. However, the risk of anastomosis breakdown is high in irradiated pelvis.

To restore or substitute urethrovesical function after cystectomy, the surgeon has different possibility: an orthotopic neobladder, supravesical urinary diversion with either continent pouches or incontinent conduits (10).

### Robotic Pelvic Exenteration Pro and Contra

In the minimally invasive pelvic exenteration, the surgeon must operate in narrow pelvic spaces, often performing dissection on fibrotic and fragile retroperitoneal tissues, previously subjected to radiation therapy or surgery. Robotic technology can help the surgeon to face these challenging conditions, particularly thanks to the 3-D vision, articulated motion, improved ergonomics, and tremor stabilization. The magnified 3D vision and articulating wristed robotic instruments may allow more accurate dissection (27).

Moreover, the robotic approach provides minimal fatigue of the operating surgeon and facilitates complex procedures such as intracorporeal suturing (22).

A 2018 meta-analysis by the Pelvex Collaborative (39) compared minimally invasive and open abdominal exenterations, showing a shorter operative time, with a median of 83 min saved, in favor of the open approach, while a reduced blood loss (median 550 vs. 2,300 ml) and shorter hospital stay (22 vs. 28 days, *p* = 0.04) were in favor of minimally invasive approach.

When the laparoscopic and robotic approaches were compared, the only difference in perioperative parameters was operative time, with a longer duration of laparoscopic surgery (27, 40). Bizzarri et al. reported a lower median operative time in the robotic group (500 vs. 660, *p* = 0.04).

Also Puntambekar et al. (22), who published a series of 10 patients undergoing anterior pelvic exenteration, reported a reduced operative time and blood loss of robotic surgery compared to their previous series of laparoscopic exenterations (41). The reduction in operative time may be related to the aid of articulating wristed robotic instruments in the close spaces of the deep pelvis.

However, the analysis of the operative time in robotics has a significant bias: some authors calculated it as the time from skin incision to the end of the surgery (docking included), others considered only the console time.

Regarding blood loss and hospital stay, our review reports a median blood loss of 365 ml (110-150) and a median hospital stay of 10.5 days 5-37)

Our review showed an estimated median blood loss that was substantially lower than those reported in the laparotomic approach by Martinez et al., ranging from 875 to 6,300 ml (42), while it was superimposable to that described in the laparoscopic series reported in the literature, varying from 160 to 500 ml (27, 41–45).

Hospital stays length was highly variable among the different studies, ranging from 17 to 33 days for the laparotomic group, and from 3.5 to 27 days in the laparoscopic one.

This variability may be explained by the differences in health care systems across different countries in which the studies were performed and by the gradual evolution of perioperative care, given that, over time, we observed a gradual shift from the laparotomic to the laparoscopic approach.

Blood loss, shorter hospital stays, and faster restoration of bowel function are the main benefits of minimally invasive surgery, although the complication rate is superimposable with the open abdominal approach (18, 39, 46). Indeed, if minimally invasive surgery may offer significant advantages in terms of perioperative outcomes its prognostic impact still has to be proven.

Although the Laparoscopic Approach to Cervical Cancer (LACC) Trial showed a difference in survival, depending on the surgical route applied in early-stage cervical cancer (47), we do not know whether the minimally invasive approach affects the prognosis even in patients with recurrent pelvic malignancy undergoing pelvic exenteration. Prospective randomized trials are currently underway to evaluate the differences between the open surgery and robotic approach in early-stage cervical cancer (48), whereas it will be challenging to design a randomized trial in the context of pelvic evisceration.

There are also some drawbacks of robotic technology that should be considered while planning a robotic pelvic exenteration. The first is the high cost of purchasing the robot, which must be added to the costs related to the use of disposable robotic instruments. In addition to the initial cost of the robotic system, the expense for each robotic instrument, which can be used only 10 times, adds significant increase to the total cost. The overall cost of robot-assisted practice is variable, depending on the requirements for each different procedure and on surgeon experience (49). Iavazzo and Gkegkes suggested that the robotic system turn cost-effective only when the surgeon overcomes the learning curve and is, therefore, able to quickly and safely complete the procedure, and if an enhanced recovery and early discharge programs are applied (50).

An additional problem in robotic surgery may be the conflict between the robotic arms due to suboptimal triangulation, if not placed at an adequate distance between each other, particularly in patients with small abdominal diameters and low BMI.

Furthermore, this should be a criterion in selecting the patients eligible for robotic pelvic exenteration.

### Resection Margin Status and Follow up

In pelvic exenteration, the most critical elements affecting survival outcomes are the lymph node status and the achievement of a free-margin resection (1, 32).

As described by Bizzarri et al. in a series of 23 patients subjected to pelvic exenteration, the free-margin resection rate of minimally invasive pelvic exenterations was 92.9% (27).

In the present review, focusing on the robotic approach, we registered a free-margin status in 88% of cases; no patient had macroscopically infiltrated margins while 6 out of 50 patients (12%) demonstrated microscopic infiltration of resection margins. In three patients the histopathological evaluation was not reported.

Also, data regarding long term follow-up are scant. Only 6 of the 13 selected studies had an available follow-up for a total of 31 patients. 19 (61.3%) patients were disease free (NED), 8 (25.8%) were dead (7 death of disease, 1 death of postoperative complication), and 4 (12.9%) were alive with disease (AWD) at 12 months of median follow up. These findings are difficult to interpret because of the small number of patients, the heterogeneity of follow-up duration and the different biology of the analyzed tumors.

Therefore, it is crucial to supplement these data with future studies, enrolling a larger number of patients with a detailed follow-up.

### Post-operative Complication

In the reported series of robotic pelvic exenterations, grade 2 postoperative complications occurred in 11 out of 51 patients (21.6%) while grade 3 were recorded in 13 women (25.5%). Only one grade 4 complications were reported (2%). Interestingly the robotic complication rate is overlapping to that reported in the laparotomic approach, ranging from 22 to 32% (32).

The probability of post-surgical complications is directly impacted by two factors: the specific type of urinary diversion and the primary bowel reanastomosis. In particular, complications related to urinary derivation were more frequent after Miami Pouch diversion. On the other hand, all major complications related to bowel resection occurred in patients who were subjected to direct colorectal reanastomosis.

Therefore, in patients with previous pelvic radiotherapy or systemic chemotherapy, probably bowel anastomosis and continent urinary reconstruction should be avoided. However, this hypothesis must be proved.

## Conclusions

Pelvic exenteration represents the very last surgical resort for diseases unresponsive to chemotherapy and already treated with pelvic radiation therapy.

In this context, a careful patient selection remains the milestone of such a mutilating surgery.

Although the introduction of the minimally invasive approach has led to advantages in terms of less blood loss and faster hospitalization time compared to the classic open surgery.

An important step forward should be to investigate the potential equivalence between minimally invasive approaches and the laparotomic one, in terms of long-term oncological outcomes.

Prospective randomized trials in this field will probably never be possible, so we still have to rely on small case series.

In this scenario, multicenter studies with larger numbers of patients and detailed follow-up information will be needed to justify such a surgical effort.

## Data Availability Statement

The original contributions presented in the study are included in the article/supplementary material, further inquiries can be directed to the corresponding author/s.

## Author Contributions

SC, MA, AF, AE, and GS: writing and data interpretation. MA, RG, ML, and GV: data analysis. MA, SC, and RG: study design and literature search. SC, MA, RG, ML, AF, FF, GV, and GS: data collection. All authors reviewing of the final manuscript.

## Conflict of Interest

The authors declare that the research was conducted in the absence of any commercial or financial relationships that could be construed as a potential conflict of interest.

## Publisher's Note

All claims expressed in this article are solely those of the authors and do not necessarily represent those of their affiliated organizations, or those of the publisher, the editors and the reviewers. Any product that may be evaluated in this article, or claim that may be made by its manufacturer, is not guaranteed or endorsed by the publisher.
